# Can quadrivalent human papillomavirus prophylactic vaccine be an effective alternative for the therapeutic management of genital warts? an exploratory study

**DOI:** 10.1590/S1677-5538.IBJU.2018.0355

**Published:** 2019-04-01

**Authors:** Hoon Choi

**Affiliations:** 1 Department of Urology, Korea University Ansan Hospital, Korea University College of Medicine, Ansan, Korea

**Keywords:** Human papillomavirus 31, Vaccines, Therapeutics

## Abstract

**Objective:**

To evaluate the treatment effect of genital warts, we investigated the quadrivalent HPV vaccine injection compared with surgical excision.

**Materials and Methods:**

This prospective study included 26 patients (M:F = 24:2) who received HPV vaccine or surgical excision. After explanation of surgical excision or HPV vaccine, 16 patients underwent surgical excision and the others received HPV vaccine injections. Based on gross findings of genital warts, treatment outcomes were classified as complete response (no wart), partial response, and failed treatment.

**Results:**

Among enrolled patients, 42% (11 / 26) patients had recurrent genital warts. In vaccination group, complete response rates of genital wart were 60% following 3 times HPV vaccine. Partial response patients wanted to excise the genital lesions before the 3 times injection, because they worried about sexual transmission of disease to their sexual partners. One patient underwent surgical excision after 3 times injection. Excision sites included suprapubic lesions, but other sites including mid-urethra and glans showed complete response after injection. At a mean follow-up period of 8.42 ± 3.27 months, 10 patients (100%) who received HPV vaccine did not show recurrence.

**Conclusion:**

The response rates after HPV vaccine injection were 90% (complete and partial). Our results suggested that HPV vaccines could be effective in management of genital warts.

## INTRODUCTION

Human papillomavirus (HPV) is the source of the most common sexual transmitted disease, which could infect the mucosa and skin of the anogenital regions. Infection with HPV causes a large proportion of uterine cervix, vaginal, vulvar, anal, and penile cancers, as well as genital warts. Completion of the HPV vaccine series is important to protect adolescents against the most common HPV types associated with cervical and penile cancers as well as genital warts before they are exposed to the virus (1). High risk HPV types, such as types 16 and 18, have been known to be the most common and carcinogenic, and these 2 HPV types were responsible for about 70% of the cases of cervical cancer (2).

Therefore, the Advisory Committee on Immunization Practices recommends HPV vaccination for girls and boys aged 11 or 12 years. Routine recommendation is quadrivalent HPV vaccine for girls and boys, or bivalent vaccine for girls at the age of 11 or 12 years (3). Quadrivalent HPV vaccine contains four HPV type-specific VLPs prepared from the L1 proteins of HPV 6, 11, 16, and 18. Bivalent HPV vaccine contains two HPV type-specific VLPs prepared from the L1 proteins of HPV 16 and 18. Both the vaccines are individually administered in a 3-dose schedule. Currently, World Health Organization recommends that adolescent girls and boys should receive 2 doses of HPV vaccine at 0 and 6 months, rather than 3 doses as reported previously, for the protection from HPV-related cancers (4). Because 2-dose schedule increases the flexibility in the interval between doses, which may facilitate vaccine uptake, HPV vaccines are recommended for girls and boys younger than 15 years of age.

Low risk HPV types, such as type 6 and 11 are the most frequent cause of genital warts (5). They are transmitted through direct skin-to-skin contact, usually during oral, genital, or anal sex with an infected partner. The treatment options for genital warts are the use of podophyllotoxin, imiquimod, or trichloracetic acid, and surgical excision (6, 7). Treatment options depend on the diagnosis, size, and location of the lesions, but local treatment could not eradicate HPV cells completely. Additionally, reduction in infectivity of HPV-related lesions by these therapeutic options is still unknown (6). With regard to the recurrence of genital warts, there is an inadequacy of controlled trials on the recurrence of warts in adults (7, 8). HPV vaccines have been known to exert protective effect on cervical cancer and genital warts, but the treatment effect is unknown. Therapeutic vaccine for HPV aims to generate cell-mediated immunity (6). Recently, some case reports showed the therapeutic effect of HPV vaccine at peri-anal and finger lesions (9, 10). We investigated the use of quadrivalent HPV vaccine injection for the effective treatment of genital warts.

## MATERIALS AND METHODS

From January 2015 to December 2016, a prospective trial was conducted on 24 male patients and 2 female patients who received HPV vaccine or underwent surgical excision. Patients were evaluated for medical history, and physical examination was conducted by routine laboratory studies before vaccination. If urethral involvement was suspected, cystourethroscopy was performed.

Recurrence of HPV was also inspected by noting the history and included 14 patients who were treatment naive, while the remaining patients had received repeated freezing, laser therapy, electrocautery, and / or other treatments.

Numerous HPV lesions within clear boundary masses with mostly wide pedicle and moist smooth surface were observed. Occasional fused lesions were also present in some patients and the majority of lesions were gray-brown in color, while a few were dark red. The HPV lesions were counted carefully. All patients were confirmed to have condyloma acuminatum by pathological review.

The potent therapeutic effect and an additional preventive effect of the HPV vaccination treatment was discussed with the patients. Then, the patients decided if they should be vaccinated initially and undergo definite treatment later if there was no response after vaccination. In cases of the patients who underwent definite surgery first, we reviewed the reason by a survey.

The HPV vaccine injection group received 3 doses (Gardasil®, Merck, Kenilworth, New Jersey) within 6 months. After first injection, the second injection was done around 2 months later and the third injection was administered around 6 months after first injection.

Surgical excision of the condyloma was performed using a surgical blade. At first, masses were excised as much as possible by blade then electrocauterization was done optionally in bleeding focus.

Treatment outcomes were assessed based on the gross findings of the condyloma lesion. After vaccine injection or surgical excision, no gross condyloma lesion was classified as complete response. The cases in which more than half of the lesions disappeared by treatment, were defined as partial response. In addition, if more than half of the lesions remained or aggravated at the follow-up visit after third injections in 6 months, the patients were classified as failed treatment.

## RESULTS

We conducted a prospective trial using 26 cases of HPV genital infection. Table-1 reveals the treatment outcomes in our cohort. Among the 26 patients, 16 underwent surgical excision and 10 were administered vaccine. The mean age of patients undergoing each of the treatment was 35.8 ± 11.2 years and 26.1 ± 6.0 years, respectively.

**Table 1:** Treatment outcome in 26 HPV genital infection cases. Sixteen patients underwent surgical excision and 10 were administered vaccine.

		Surgical excision (n=16)	HPV vaccine injection (n=10)	P - value
Base line characteristics				
**Sex**				
	M/F	15/1	9/1	0.064*
	Age (year)	35.8±11.2	26.1±6.0	0.016†
**Condyloma recurrent history**				**0.076** †
	None	11 (68.75%)	4 (40%)	
	More than once	5 (31.25%)	6 (60%)	
**Condyloma counts**				**0.242†**
	1	3 (18.75%)	1 (10%)	
	2-4	3 (18.75%)	5 (50%)	
	≥ 5	10 (62.50%)	4 (40%)	
**Treatment response**				
Complete response after injection		Not assesed	6 (60%)	
Partial response after injection		Not assesed	1	
No response			3	

* Mann-Whitney test

† Fisher exact test

About 42% of the patients had a history of genital warts that recurred more than once. Absolute results of recurrent infection were 60% in vaccine injection patients and 31.25% in surgical excision patients. In case of the HPV lesion, only one condyloma was counted in 18.75% / 30%, two to four in 18.75% / 30%, more than five in 62.50% / 40% in surgical excision and vaccine injection patients, respectively.

**Figure 1:**
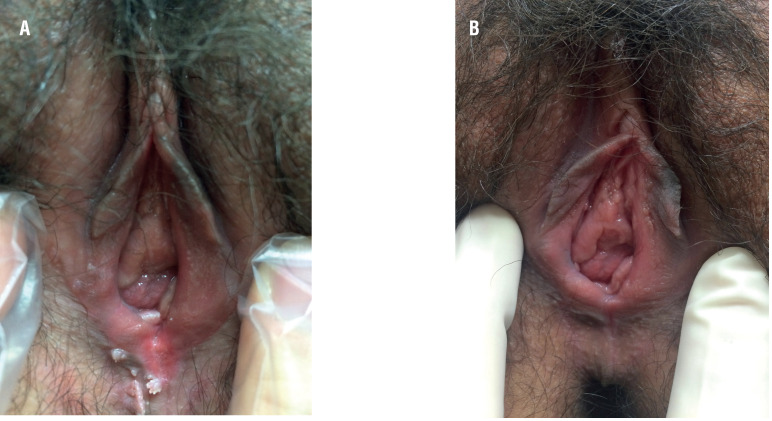
(a) Multiple HPV lesions on the external genitalia of a 36-year-old female patient; and (b) the HPV lesions disappeared completely after vaccination.

Complete response rates of genital wart were observed to be 60% following 3 HPV vaccine injections at a mean follow-up period of 8.42 ± 3.27 months. In a case of complete response by vaccination, a 36-year-old female patient had multiple HPV lesions on the external genitalia and around the urethral meatus as shown in Figure-1a, and the HPV lesions disappeared completely as shown in Figure-1b. After three vaccinations, partial response patients wanted to excise the genital lesions before the 3 injections, because they were worried about sexual transmission of the disease to their sexual partners. Three patients with no response underwent surgical excision after 3 HPV vaccine injections.

Among 16 patients who underwent surgical excision, 8 (43.75%) had expensiveness as the major concern, followed by 3 who were worried about treatment failure (18.75%), 2 worried about sexual transmission (12.5%), 2 worried about side effects (12.5%), and one patient did not answer the question.

## DISCUSSION

HPV is a DNA virus from the papillomavirus group with more than 170 family types (11). HPV resides in the basal cells of the stratiﬁed squamous epithelium and squamocolumnar junction of the cervix. Therefore, systemic immunological reaction may be prohibited.

Most of HPV infections are temporary and asymptomatic and raise no medical problems. Seventy percent of new HPV infections are self-limited within 1 year, and almost 90% are cleared within 2 years.

The duration of new infections is 8 months in average (12). The risk for persistence and development to precancerous lesions is designated by HPV type, with HPV 16 being more aggressive than other high-risk HPV subtypes. Issues related to cancer progression in epidemiologic data were smoking, increased age, history of sexually transmitted infections, immunologic suppression, and other host factors. The time between primary HPV infection and progress of cancer usually take decades (13).

Irrespective of the localization, HPV invades until the glandular epithelium of the cervix resulting in glandular cancers, such as carcinoma in situ or invasive adenocarcinoma (14). The HPV vaccine is intended to prevent people from getting infected with the virus, but in some cases, it may actually work as a treatment, clearing warts in people who are already infected. In the United States, about 47 million new HPV outbreaks occurred every year and caused considerable personal morbidity and social burden (15). Until now, owing to improvement in topical therapeutic approaches, surgery can be circumvented in several cases (16). Nonetheless, surgical intervention is the representative treatment in most of the cases, especially, in high risk condyloma or if regional therapy does not seem viable owing to the unfavorable size or location of the affected site.

The natural history of naturally acquired immunity related to HPV is poorly understood. HPV vaccine has been valid for routine vaccination of juvenile girls since 2006. It is true that HPV vaccination has been mainly focused on young female patients. Irrespectively, HPV vaccine has been proven to also be useful in male patients for the prevention of HPV recurrence and its associated malignancies. Recently, HPV vaccine has been permitted to be used for male patients in several countries as well (17, 18). The Brazilian Ministry of Health does not recommend that as a public health guideline, but the Brazilian Agency of Sanitary Surveillance (ANVISA) indicates that vaccination in women aged 11 to 26 years (19).

Consequently, HPV vaccine has been shown to be potent in preventing genital warts and related cancers in both genders. Vaccine is a medical agent that generates antibody and provokes a subclinical immunological effect in the human body by enhancing immunity to the specific infection. HPV creates warts in various proportions of the cervix, vagina, and vulva in female patients; the penile and scrotum areas in male patients; as well as the anal area in both genders. Therefore, completion of the HPV vaccination series is essential to protect patients against the most common HPV types associated with genital warts previous to the exposure of HPV.

Recently, the idea of vaccination was extended to the concept of its therapeutic usage. Sustained expression of the HPV oncoproteins, E6 and E7 are crucial factors in the development of intraepithelial neoplasia and cancers, and thus, creating main targets for therapeutic vaccines (20).

In the cervical HPV infection, complete response of cutaneous warts was noted after 3 injections of the vaccine (21). Another patient with exophytic wart masses on the perianal area had dramatic regression after a single course of HPV vaccination (9). However, most clinical trials of vaccines against HPV-related diseases have focused on pre-cancers and cancers of the cervico-vaginal area.

Even though HPV vaccine was intended to produce neutralizing antibodies to avoid reinfection, its abilities to induce cell-mediated immunological responses were confirmed in previous studies (22, 23). Additionally, adoptive T cell transfer therapy has been recently evaluated in patients with metastatic cervical cancer, and the proportion of clinical response with HPV-specific T cells was 33%; the case reports providing significant evidence that HPV-related disease can be susceptible to T cell-mediated mechanisms are limited (24). Therefore, the rapid therapeutic reaction of HPV vaccination had relevance in the activation of the immunological system, including T cells and macrophages.

Some previous clinical trials assessing the outcomes of therapeutic HPV vaccinations showed unsatisfactory results, and studies on therapeutic vaccines against HPV are mainly being published as case reports as discussed above (25). On the contrary, experimental trials with positive effect were also conducted. Fifty-four female patients with high grade cervical intraepithelial neoplasia (CIN) were administered MVA E2 (vaccinia virus Ankara containing the bovine papilloma virus E2 protein) therapeutic vaccine. Total elimination of high-grade lesions was observed in 20 out of 34 patients with MVA E2 vaccine. Eleven patients showed reduction in size by more than half and in three other patients, the warts were decreased to CIN 1 or 2 (26).

Recent experimental study on the combined use of the imiquimod 5% cream and quadrivalent recombinant HPV vaccination to achieve long-term clinical remission of chronic HPV infection manifested by anogenital warts was published. This study enrolled 36 young patients (22 men) that were vaccinated by quadrivalent recombinant and co-administered with imiquimod 5% cream three times in a week up to four months. Complete remission of genital warts was observed in 94.4% patients within 1 year and 2 patients with anogenital warts were treated with Solcoderm® cream which completed the removal of genital warts. There were no recurrences throughout follow-up period (27).

Another report showed noninferiority of the immune reaction in males (age 10-15 years) compared with same aged female group, and in males (age 9-15 years) compared with same females group it was demonstrated that both geometric mean titer and seroconversion rates for all four HPV vaccine types met non-inferiority criteria in the per-protocol population. So we may expect the similar treatment effect irrespective of gender (28).

Our case series is the first to reveal the therapeutic effect of HPV vaccination. The response rates of 90% (complete and partial) after vaccine injection were promising. Besides, our idea of therapeutic vaccination on the genital warts is based on some valuable merits. Firstly, genital warts are not a fatal disease, so we can choose a treatment from multiple options considering various factors, and definite surgical treatment could be deferred after systemic vaccinations. Additionally, therapeutic vaccination is relatively non-invasive, because surgical management may induce pain and scar formation. Furthermore, we should be cautious about viral seeding during ablation procedure. If HPV infects the cervix, approximately 90% of the infections are cleared without any management within 2-3 years even though variation depends on their type and risk of HPV. Cellular immunity plays an important role in this natural loss of HPV infection (29, 30). This means that immunological aspect is crucial for therapeutic clearance of HPV infections, with vaccination occupying important part in preventive advantages. Lastly, HPV does not invade the epithelium and systemic defensive reaction by antibody cannot be induced. Thus, major preventive advantage by therapeutic vaccination is definitive compared with that by curative local management (12). Though our successful data on complete response on vaccination treatment could be merely reflection on the tendency toward faster resolution of HPV infections, no one can conclude that therapeutic vaccinations are valuable medical therapy or just secondary assistant management with minimal merits.

HPV vaccines as a treatment option have to overcome several hurdles. Therapeutic vaccination has not been officially evaluated in a randomized prospective trial and accordingly it has not received a formal approval in any country. Therefore, there remains a critical necessity for clinical trials to evaluate the efficacy of HPV vaccination as a treatment. We need to understand the difference in clinical response of patients after vaccination for treatment of HPV infection. Various concerns of patients who underwent curative resection, such as expensive cost, treatment failure, sexual transmission, should be overcome by cost analysis or education about the barriers during the treatment period.

Some patients who were administered with HPV vaccine for the management of genital warts, were worried about sexual transmission to their partners. They underwent surgical excision during the vaccine administration period and the infection did not recur during the follow-up. So, to maximize the efficiency of the vaccine, the possible candidates should be vaccinated irrespective of the gender and condition of the patients.

This research has several limitations, such as small number of patient group and no prospective randomized control group. Additionally, we do not have HPV typing result and biomarkers for immunological responses or risk level. We explained potent therapeutic effect of vaccine that was not fully proven for genital warts. So this may introduce a very strong bias and misinformation in the consent process. Patients were mixed with the first episode and recurrent episode, so the immunology issues may be different in both cases. Furthermore, we do not know the optimal dose or regimen enough to protect and we should not know the age-related treatment option even many countries are not limiting the age for people to get the prophylactic vaccine.

Even though our study has some drawbacks, our data on therapeutic vaccination shows its potential benefit as an excellent treatment candidate to motivate clinical regression in genital warts.

Genital warts remain a serious issue worldwide because early diagnosis is difﬁcult and proper primary management is sometimes missing. In the absence of vaccine coverage, the presence of high number of HPV infections, and the incidence of HPV-related tumors will remain a global public concern worldwide, including Korea. In conclusion, classical concept of vaccination is to generate beneficial immunological effect in the body to prevent recurrences. In our opinion, therapeutic HPV vaccination can eliminate genital warts, leaving people with better protection against future recurrences caused by HPV relapse.

## CONCLUSIONS

The response rates of our research after therapeutic HPV vaccination indicated effectiveness as an initial management approach of genital warts with potent preventive advantages over recurrent infection.
